# A symptomatic anterior mediastinal mass with a simultaneous *Haemophilus influenzae* infection

**DOI:** 10.1002/rcr2.394

**Published:** 2018-12-04

**Authors:** Hui Zen Hee, Chun‐Ku Chen, Yi‐Chen Yeh, Chien‐Sheng Huang

**Affiliations:** ^1^ Division of Thoracic Surgery, Department of Surgery Taipei Veterans General Hospital Taipei Taiwan; ^2^ School of Medicine, National Defense Medical Center Taipei Taiwan; ^3^ Department of Radiology Taipei Veterans General Hospital Taipei Taiwan; ^4^ Department of Pathology Taipei Veterans General Hospital Taipei Taiwan; ^5^ Institute of Clinical Medicine, School of Medicine, National Yang‐Ming University Taipei Taiwan

**Keywords:** *Haemophilus influenzae* infection, lung abscess, mature mediastinal teratoma

## Abstract

Surgical resection remains the treatment of choice for mature mediastinal teratoma, and the operation itself is sometimes complicated or life‐threatening, especially when it ruptures into adjacent vital structures. We describe a rare case of unanticipatedly delayed complete resection of a symptomatic teratoma with simultaneous *Haemophilus influenzae* infection, followed by extended rupture into the pleural space, lung, and bronchus. The clinical presentation and the microbiological and radiologic features may lead to the impression of a lung abscess until it can be proven otherwise pathologically after an initial thoracic aspiration. Accordingly, surgical intervention through a minimal approach, such as video‐assisted thoracoscopic surgery, might be considered a strategy after the initial extended rupture.

## Introduction

Mature mediastinal teratoma (MMT) is usually asymptomatic and found incidentally 1. Under chest computed tomography (CT) imaging, a mature teratoma typically appears as a heterogeneous mass with soft tissue content, calcification, cystic fluid, fat, or any of the above combinations 2. Misdiagnosis of a benign MMT is not unusual, particularly in the ruptured cases, which represent up to 36–41% of all cases 3. Severe symptoms such as haemoptysis and chest pain are more common in ruptured tumours 4. Surgical resection of the tumour remains the treatment of choice for unruptured MMTs. However, the optimal timing of surgical resection for ruptured MMTs is still not well defined. We report a rare case of *Haemophilus influenzae*‐infected MMT with extended rupture into the pleural space, lung, and bronchus, followed by subsequent regressive change and a successful resection through video‐assisted thoracoscopic surgery (VATS).

## Case Report

**Figure 1:**
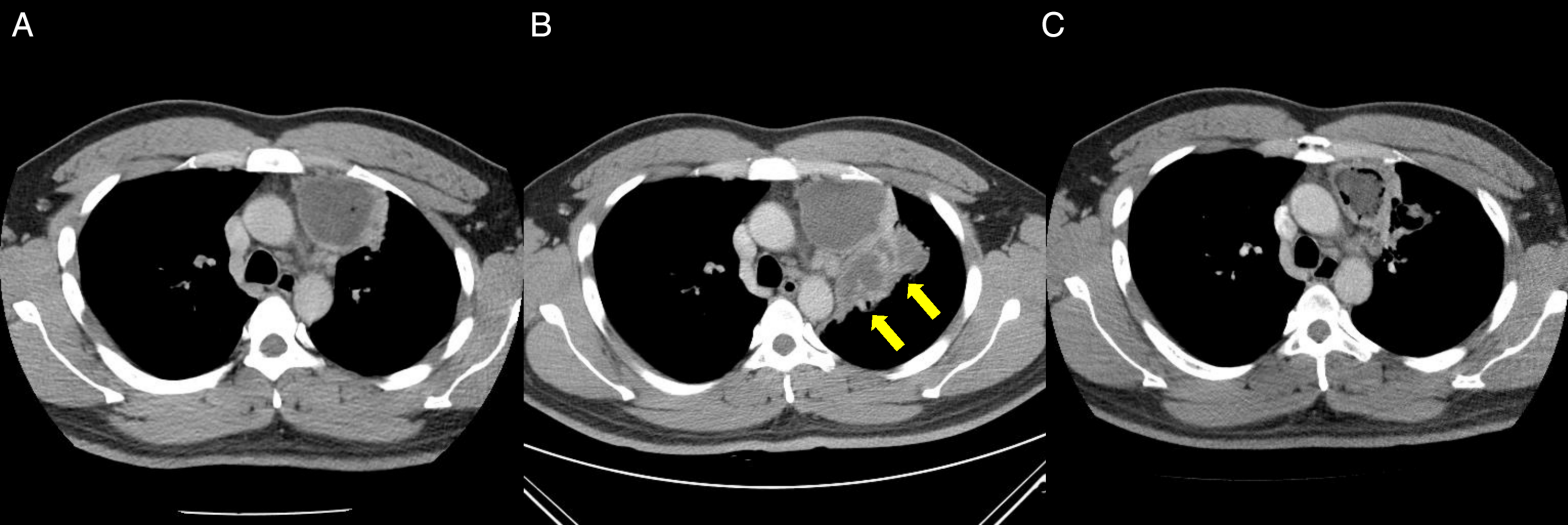
Axial post‐contrast chest computed tomography of a 35‐year‐old man. (A) A cystic heterogeneous lesion over the upper left hemithorax, measuring 7.0 × 6.0 × 5.0 cm. (B) Three weeks later, the lesion enlarged and showed multiloculation, with minimal air bubble sand perforating into the lung and bronchus (arrows). (C) Seven months later, the lesion showed shrinkage in size with irregular consolidation at the upper left lobe of lung.

**Figure 2:**
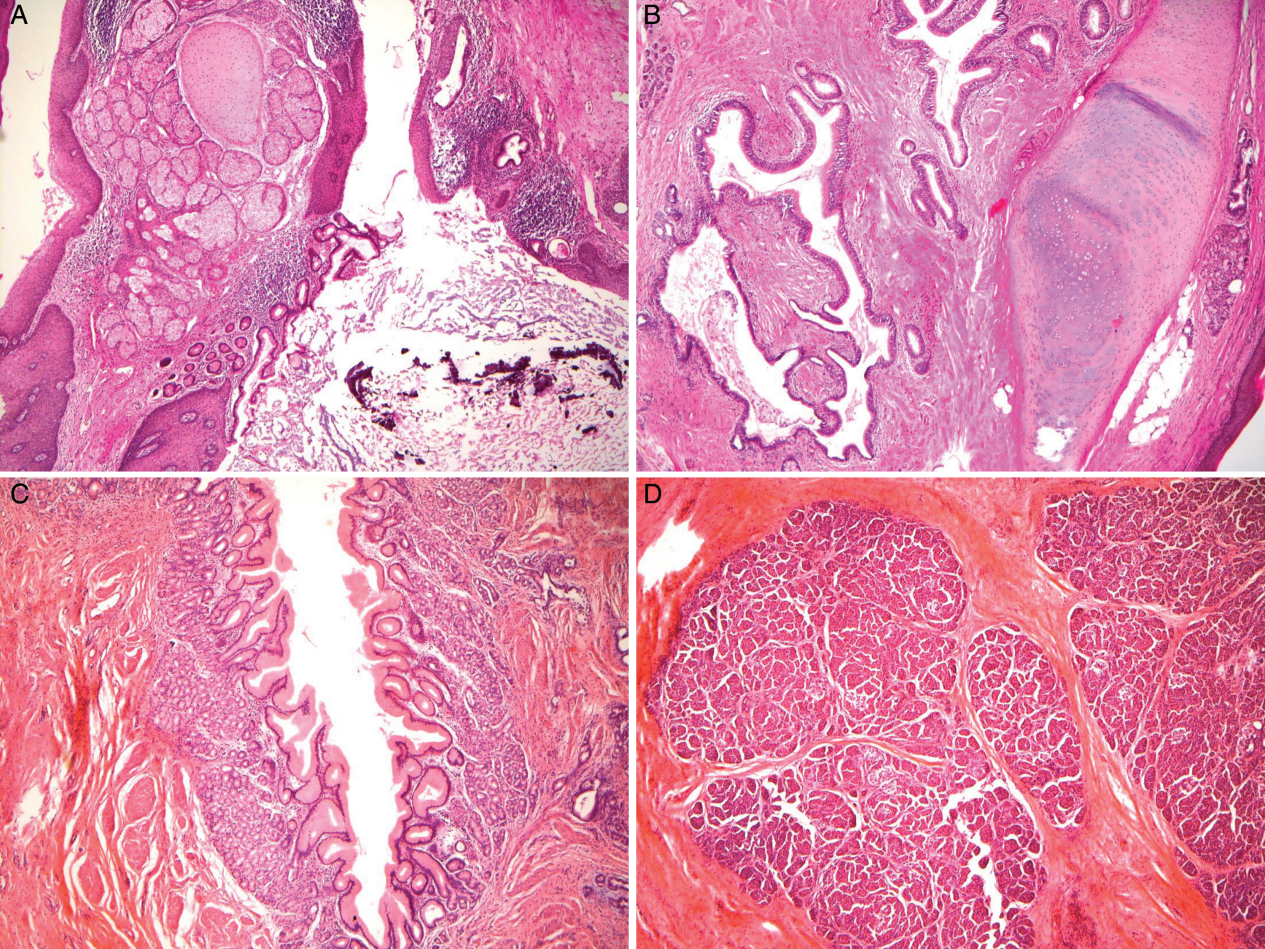
Histological examinations showed that the pathological features of mature cystic teratoma include (A) mature epidermis, skin appendages; (B) cartilage, adipose tissue, and respiratory epithelium; (C) gastric‐type mucosa; and (D) pancreatic tissue. (original magnification: 40×).

A 35‐year‐old, non‐smoking man suffered from coughing with blood‐tinged sputum for three months and eventually developed intermittent low‐grade fever. He mentioned that the cough was episodic, consuming, with mild reddish‐brown sputum, and had progressed over the last month. He was admitted to the local hospital, and a CT scan demonstrated a cystic heterogeneous lesion over the left upper lobe (LUL) of the lung, measuring 7.0 × 6.0 × 5.0 cm, in the left upper paramediastinal region (Fig. 1A). Under the impression to rule out the possibility of malignancy with necrosis, ultrasound‐guided fine‐needle aspiration of the mass was arranged and demonstrated pus‐like material. The culture was positive for *H. influenzae*, and the cytology was negative for malignant cells. His cough persisted despite antibiotic treatment for three weeks with Augmentin, Cefuroxime, Ciprofloxacin, and Cefepime, each over a course of 5–7 days. A CT scan was again arranged and disclosed a progressively extending multiloculated, thick‐walled cystic lesion with minimal air content cavity lesions at the left apical lung with pleural effusion (Fig. 1B). Bronchoscopy showed no evidence for endobronchial lesions or malignant cells upon cytology. The patient was then transferred to our hospital for further treatment. Ultrasound‐guided aspiration was repeated and demonstrated a hypoechoic mass lesion at the upper left lung field with multiple cystic changes that was negative for malignant cells, and neither bacterial, mycobacterial, nor fungal growth was detected at this time. The patient refused surgical intervention and was discharged two weeks later in an ameliorated state. After that, he was scheduled three times for an outpatient department (OPD) follow‐up, and plain chest films exhibited regression of the previous lesion. The patient did not present any specific complaints. Seven months after his last OPD visit, he developed haemoptysis again, and this time, the CT scan demonstrated a residual thick‐walled cavitary lesion 7.0 × 5.0 cm in size (Fig. 1C), suggesting a residual organizing lung abscess at the left upper lung with newly found bronchiectasis. Due to the persisting lesion and failure of the medical treatment, the thoracic surgeon was consulted, and we performed a left VATS. This indicated a tumour mass located in the anterior mediastinum with severe adhesion to the aortic arch, left main pulmonary artery root, phrenic nerve, and LUL. The resected mass measured 7.0 × 5.0 × 4.0 cm. It was encapsulated and demonstrated a cystic component containing hairs and sebum on sectioning. Microscopically, the specimen was compatible with mature cystic teratoma with mature epidermis, skin appendages, respiratory epithelium, pancreatic tissue, gastric‐type mucosa, cartilage, and adipose tissue (Fig. 2). The patient had an uneventful postoperative recovery and was discharged on the fourth postoperative day. He remained well afterwards and on 3‐year follow‐up. The patient’s written informed consent for publication was obtained.

## Discussion

This case is unique in that its initial presentation and further investigation mimicked a lung abscess and in the demonstration of a regressive change of a ruptured MMT. A subsequently suspended resection through VATS successfully enhances the distinctiveness of this case.

Mature teratomas are usually asymptomatic 1. While symptoms such as haemoptysis and chest pain are more common in ruptured tumours 4, in this case, the CT obtained at the patient’s initial presentation did not show any rupture of the cystic lesion, and this was coupled with pus‐like material and positive bacterial growth from the aspirated fluid. Infected teratomas in adults are rare, and while a case of *H. influenzae* 5 and *Mycoplasma pneumoniae* 6 infection were each reported in ruptured teratomas, the infection of un‐ruptured teratomas was not reported previously.

The transformation of the radiological features of this case contributes to an interesting discovery that was not previously reported in MMTs. The second CT image obtained three weeks after the patient’s initial image was taken showed a progressive change into a ruptured lesion. Although several mechanisms of rupture in MMT have been proposed 3, we speculated that the tumour mass aspiration that was performed could partly have induced the rupture. This leads to another topic that is not further discussed in this literature, which is whether an aspiration should be performed before a teratoma is completely ruled out.

To our knowledge, regression of the lesion with the amelioration of clinical symptoms has not been previously described in adult MMTs, especially when rupture into adjacent structures occurs as a complication. Surgical resection of the tumour remains the treatment of choice for MMTs. For unruptured MMTs, early surgery is usually recommended because of the risk of rupture, the development into serious and life‐threatening complications, or malignant transformation 3. In contrast, the optimal timing of surgical resection for a ruptured MMT is still undetermined. Delayed surgical resection of a ruptured MMT might be more complicated due to its digestive autolysis and chemical, ischaemic, and/or necrotic sequelae. However, the surgical intervention itself is sometimes complicated or life‐threatening, especially when there is a rupture into adjacent vital structures. Jothianandan et al. reported a ruptured case presenting with the impossibility of complete initial resection via median sternotomy because the tumour had infiltrated into the pericardium and encased both ventricles and the coronary arteries. The patient was still doing well after 12 months of follow‐up 5. Suwatanapongched et al. reported the intrapulmonary rupture of an MMT that presented with active bleeding during surgery via thoracotomy with a median sternotomy approach due to the tight adherence to the anterior segment of the right upper lobe 3. Indeed, there are currently no clinical guidelines to adopt a delayed resection strategy of a ruptured MMT. To avoid massive bleeding or incomplete resection, preoperative planning regarding the resectability of a ruptured MMT should be considered carefully. Furthermore, our case demonstrates the practicality of postponing the resection of a ruptured benign MMT through a minimally invasive approach after a regressive change.

In conclusion, this case report highlights the possibility of an infected MMT with impending rupture despite initial investigations suggesting a lung abscess. An early recognition and surgical resection of an infected MMT might spare the complication of rupture into the lung, pleural space, or pericardial space. Furthermore, according to our report, suspended delay surgical intervention through a minimal approach, such as VATS, might be a strategy to consider for its initial extended rupture.

### Disclosure Statement

Appropriate written informed consent was obtained for publication of this case report and accompanying images.

## References

[1] Duwe BV , Sterman DH , and Musani AI . 2005 Tumors of the mediastinum. Chest 128:2893–2909. 16236967 10.1378/chest.128.4.2893

[2] Moeller KH , Rosado‐de‐Christenson ML , and Templeton PA . 1997 Mediastinal mature teratoma: imaging features. Am. J. Roentgenol. 169:985–990. 9308448 10.2214/ajr.169.4.9308448

[3] Suwatanapongched T , Kiatboonsri S , Visessiri Y , et al. 2011 A 30‐year‐old woman with intermittent cough and a mass‐like opacity in the right upper lobe. Chest 140:808–813. 21896526 10.1378/chest.10-2292

[4] Choi SJ , Lee JS , Song KS , et al. 1998 Mediastinal teratoma: CT differentiation of ruptured and unruptured tumors. Am. J. Roentgenol. 171:591–594. 9725279 10.2214/ajr.171.3.9725279

[5] Jothianandan K , Tibb AS , McLemore M , et al. 2010 An adult man presenting with hemoptysis caused by mature teratoma with rupture into the bronchus and pericardium and complicated by *Haemophilus influenzae* infection. J. Thorac. Cardiovasc. Surg. 139:e104–e107. 19660363 10.1016/j.jtcvs.2009.03.003

[6] Yu CW , Hsieh MJ , Hwang KP , et al. 2007 Mediastinal mature teratoma with complex rupture into the pleura, lung, and bronchus complicated with *Mycoplasma pneumonia* . J. Thorac. Cardiovasc. Surg. 133:1114–1115. 17382673 10.1016/j.jtcvs.2007.01.006

